# Mechanisms underlying HIV-1 Vpu-mediated viral egress

**DOI:** 10.3389/fmicb.2014.00177

**Published:** 2014-05-01

**Authors:** Nicolas Roy, Grégory Pacini, Clarisse Berlioz-Torrent, Katy Janvier

**Affiliations:** ^1^INSERM U1016, Institut CochinParis, France; ^2^CNRS UMR8104Paris, France; ^3^Université Paris DescartesParis, France

**Keywords:** Vpu, CD4, BST2/Tetherin, HIV-1, ESCRT, degradation, cell surface down-regulation, NF-κB

## Abstract

Viruses such as lentiviruses that are responsible for long lasting infections have to evade several levels of cellular immune mechanisms to persist and efficiently disseminate in the host. Over the past decades, much evidence has emerged regarding the major role of accessory proteins of primate lentiviruses, human immunodeficiency virus and simian immunodeficiency virus, in viral evasion from the host immune defense. This short review will provide an overview of the mechanism whereby the accessory protein Vpu contributes to this escape. Vpu is a multifunctional protein that was shown to contribute to viral egress by down-regulating several mediators of the immune system such as CD4, CD1d, NTB-A and the restriction factor BST2. The mechanisms underlying its activity are not fully characterized but rely on its ability to interfere with the host machinery regulating protein turnover and vesicular trafficking. This review will focus on our current understanding of the mechanisms whereby Vpu down-regulates CD4 and BST2 expression levels to favor viral egress.

## INTRODUCTION

Viral egress and replication rely on a complex interplay between viral and cellular proteins. During their replication cycle, viruses, notably lentiviruses, have to face several levels of the host immune defense mechanisms and must counteract these barriers to persist and disseminate in the host. Understanding the mechanisms underlying lentiviruses evasion from host antiviral activities has been the focus of many studies of the past decades. Not only did they contribute to major advances in the characterization of host strategies to repress viral replication, notably by the identification of antiviral cellular proteins such as APOBEC3G, SAMHD1, TRIM5α, and BST2 referred to as restriction factors (Sheehy et al., 2002; Stremlau et al., 2004; Neil et al., 2008; Van Damme et al., 2008; Laguette et al., 2011), but also they unraveled the importance of the accessory proteins of viruses in this process (Malim and Bieniasz, 2012).

The genome of lentiviruses encodes for several accessory proteins such as Nef, Vif, Vpr, Vpx, and Vpu, in addition to the structural and enzymatic proteins Gag, Pol, and Env and the regulatory proteins Tat and Rev (Malim and Bieniasz, 2012). These accessory proteins are, however, not common to all lentiviruses: Nef and Vpr are specific of primate lentiviruses (HIV-1, HIV-2, and SIV), Vpx is expressed by HIV-2 and its closely related SIV_smm_ and SIV_mac_, Vpu is expressed by HIV-1 strains and a few strains of SIV (described later; Malim and Emerman, 2008). These proteins are not strictly required for viral replication *in vitro*. However, much evidence has highlighted their importance in the pathogenesis of the infection, as they contribute to modify the cell environment to facilitate viral replication and evasion from the host antiviral immune response (Malim and Emerman, 2008).

This review will provide an overview of our current understanding of the mechanisms whereby the accessory protein Vpu exploits the host cell machineries to counteract two components of the adaptive and innate host immune system: the protein CD4 and the restriction factor BST2.

## CHARACTERISTICS AND FUNCTIONS OF THE ACCESSORY PROTEIN VPU

The accessory protein Vpu is an 81-amino acid type I integral membrane phosphoprotein, expressed by the genome of HIV-1, the related SIV_cpz_ (Strebel et al., 1988) and the SIV_gsn_ lineages including the greater spot-nosed monkey (SIV_gsn_), the mona monkey (SIV_mon_), the mustached monkey (SIV_mus_) and Dent’s mona monkey (SIV_den_) isolates (Gao et al., 1999; Courgnaud et al., 2002, 2003; Bailes et al., 2003). Vpu contains a short luminal N-terminal domain, a 23-amino acid transmembrane domain and a large cytoplasmic C-terminal domain (Strebel et al., 1988; Maldarelli et al., 1993). Vpu localizes mainly in the endoplasmic reticulum (ER), the *trans*–Golgi network (TGN) and endosomal compartments. Two major functions have been attributed to Vpu during HIV-1 replication cycle. Firstly, Vpu targets the newly synthesized CD4 receptor for proteasomal degradation (Willey et al., 1992a). Secondly, it favors the release of viral particles from most human cell types through counteracting the inhibitory effect of BST2 (Neil et al., 2008; Van Damme et al., 2008). More recently, Vpu was shown to down-regulate cell surface expression of two additional mediators of the immune response: the lipid-antigen presenting protein CD1d expressed by antigen-presenting cells (Moll et al., 2010) and the natural killer cells ligand NTB-A (Shah et al., 2010). Vpu appears therefore as a key factor for HIV evasion from the host immune system.

## VPU-INDUCED DOWN-REGULATION OF CD4

CD4 constitutes the major component of the receptor complex used by primate lentiviruses to infect the cells. It is a 54 kDa type I integral glycoprotein expressed at the surface of helper T-lymphocytes, cells of the monocyte/macrophage lineage and hematopoietic progenitor cells.

Infection of CD4^+^ cells by primate lentiviruses results in a rapid and constant down-modulation of cell surface CD4 expression level (Ray and Doms, 2006). CD4 down-regulation was proposed to prevent lethal superinfection of cells by additional virions (Wildum et al., 2006), contribute to the escape of infected cells from the immune system and favor viral fitness (Willey et al., 1992b). CD4 depletion in infected cells is achieved by the concerted, though mechanistically distinct, action of three viral proteins: Nef, Vpu and, to a lower extent, Env (Wildum et al., 2006). Nef, produced shortly after infection, enhances internalization of pre-existing CD4 from the cell surface and targets the receptor for lysosomal degradation (Chaudhuri et al., 2007). Env precursor gp160 binds CD4 in the ER and blocks its transport to the cell surface (Crise et al., 1990; Jabbar and Nayak, 1990). Vpu targets CD4 molecules present in the ER for proteasomal degradation (Willey et al., 1992a).

**FIGURE 1:**
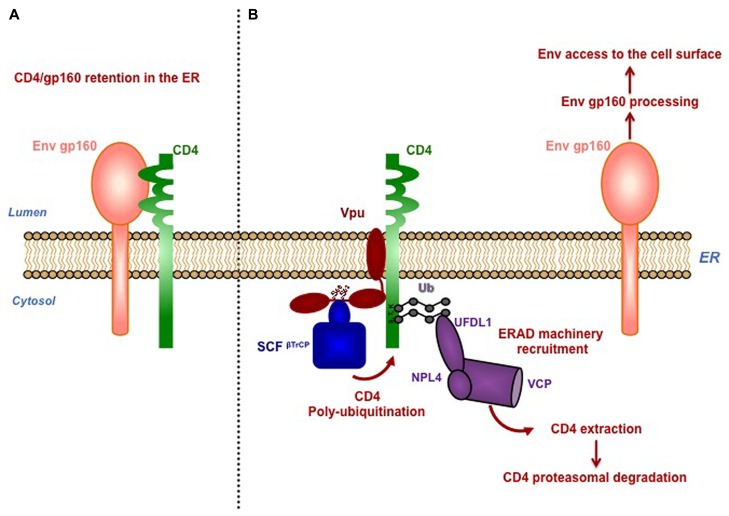
**Vpu mediates proteasomal degradation of CD4 to favor viral fitness. (A)** Newly synthetized CD4 and HIV-1 envelope precursor gp160 interact in the ER through their luminal domain, preventing Env trafficking to the cell surface. **(B)** Vpu induces retention of CD4 in the ER through interaction *via* their transmembrane region and connects CD4 to SKP1-cullin1-F-Box (SCF) E3 ubiquitin ligase through binding with the SCF subunits β-TrCP1 and β-TrCP2. Interaction of Vpu with β-TrCP involves the conserved phophorylated serines S_52_ and S_56_, located in the cytoplasmic tail of Vpu. Recruitment of the SCF^β-TrCP^ complex by Vpu induces poly-ubiquitination of CD4 on lysine, serine and threonine residues in its cytoplasmic tail. Poly-ubiquitination of CD4 partially contributes its retention in the ER, through a yet-to-be-determined mechanism. Vpu-induced SCF-mediated poly-ubiquitination of CD4 enables the recruitment of the ERAD VCP-UFD1L-NPL4 dislocase complex, leading to the extraction of CD4 from the ER membrane and its subsequent degradation by the proteasome. Degradation of CD4 by Vpu was proposed to dissociate CD4 from newly synthesized viral Env present in the ER, allowing Env maturation and trafficking to the cell surface for its subsequent incorporation in the forming virions.

Vpu induces degradation of newly synthesized CD4 by a multi-step process involving binding of Vpu with CD4 *via* their transmembrane domains (TMD), retention and poly-ubiquitination of CD4 in the ER, followed by its delivery to the ER-associated degradation pathway (ERAD) for further proteasomal degradation (Magadan et al., 2010; Magadan and Bonifacino, 2012; **Figure 1**). Vpu-induced degradation of CD4 requires the integrity of two phosphoserines S_52_/S_56_ present in a canonical DSGXXS motif within the cytoplasmic tail of Vpu and involved in an interaction with the β-transducin repeat-containing protein 1 or 2 (β-TrCP1; β-TrCP2), two adaptors for the SKP1-cullin1-F-Box (SCF) E3 ubiquitin ligase complex (Margottin et al., 1998). Recruitment of the SCF^β-TrCP^) complex by Vpu results in poly-ubiquitination of the cytoplasmic tail of CD4 on lysine, serine and threonine residues (Magadan et al., 2010). Interestingly, Vpu-induced SCF-mediated poly-ubiquitination of CD4 contributes to retain the receptor in the ER and enables the recruitment of the ERAD VCP-UFD1L-NPL4 dislocase complex, leading to the extraction of CD4 from the ER membrane and its subsequent degradation by the proteasome (Magadan et al., 2010; **Figure 1**).

## VPU-MEDIATED ANTAGONISM OF THE RESTRICTION FACTOR BST2

A major breakthrough in understanding how Vpu promotes the release of HIV-1 particles was made by the identification of BST2 as a restriction factor for HIV-1 release (Neil et al., 2008; Van Damme et al., 2008).

### CHARACTERISTICS OF BST2

BST2 is a 30–36 kDa highly glycosylated type II integral membrane protein, constitutively expressed in several cell types and can be up-regulated by type-I interferon and pro-inflammatory stimuli (Neil, 2013). BST2 is composed of a short N-terminal cytoplasmic tail, linked to a transmembrane domain and an extracellular domain anchored to the membrane through a C-terminal glycosyl-phosphatidylinositol (GPI) moiety (Kupzig et al., 2003). Recently, a short isoform of BST2 produced by an alternative translation initiation from the methionine residue at position 13 has been identified (Cocka and Bates, 2012). BST2 is localized at the plasma membrane (PM) in cholesterol–rich microdomains (rafts) and in intracellular compartments such as the TGN as well as early and recycling endosomes (Kupzig et al., 2003; Masuyama et al., 2009). BST2 was proposed to assemble as a “picket fence” around the lipid rafts, playing a role in organizing membrane microdomains (Billcliff et al., 2013). BST2 was shown to physically trap the *de novo* formed mature viral particles at the surface of infected cells, thereby considerably reducing virus release (Neil et al., 2008; Van Damme et al., 2008; Perez-Caballero et al., 2009; Hammonds et al., 2010). This activity relies on BST2 ability to form parallel disulfide–bond homo-dimers and to bridge virions and cellular membranes *via* its N– and C–terminal membrane anchoring domains (Iwabu et al., 2009; Perez-Caballero et al., 2009; Schubert et al., 2010), with a preference for an “axial” configuration in which the GPI anchors are inserted into virions, and the N-termini transmembrane anchors remain in the infected cells membrane (Venkatesh and Bieniasz, 2013).

Although initially identified as the factor responsible for defective release of HIV-1 mutants lacking the accessory gene *vpu* (Neil et al., 2008; Van Damme et al., 2008), it is now well established that BST2 restricts the release of nearly all enveloped viruses (retroviruses, herpes viruses, filoviruses, rhabdoviruses, paramyxoviruses, and arenaviruses) (Neil, 2013). BST2 therefore appears as a major mediator of the innate immune defense against viral dissemination. Primates lentiviruses deploy three proteins to antagonize BST2 antiviral activity: Vpu in HIV-1 (Neil et al., 2008; Van Damme et al., 2008); Env in HIV-2 ROD10, HIV-2 RODA, SIV_agm_Tan and SIV_mac239_Δ*nef* isolates (Gupta et al., 2009b; Le Tortorec and Neil, 2009; Hauser et al., 2010; Serra-Moreno et al., 2011) and Nef in most isolates of SIV (Jia et al., 2009; Sauter et al., 2009; Zhang et al., 2009). HIV-1 Vpu, HIV-2 Env, and SIV Nef were shown to down-regulate the cell surface expression level of BST2 to favor its removal from viral budding sites and further viral release (Neil, 2013). To date, the precise mechanism involved in this process has not been fully characterized.

### BINDING OF VPU WITH BST2

Binding of Vpu with BST2 through their respective TMD was shown to be essential to counteract BST2 antiviral activity. Mutagenesis analyzes have unraveled the critical role of residues I_34_, L_37_, L_41_ of BST2 and A_14_, W_22_ and to some extent A_18_ of Vpu in this interaction (Vigan and Neil, 2010; Kobayashi et al., 2011). These residues were proposed to form an anti-parallel helix-helix interface (Skasko et al., 2012). Interestingly, recent studies have identified additional residues, located at the periphery of the TMD of BST2 and Vpu respectively, required for the interaction between both proteins and antagonism of BST2 (McNatt et al., 2013; Pickering et al., 2014).

### VPU-INDUCED CELL SURFACE DOWN-REGULATION OF BST2

**FIGURE 2:**
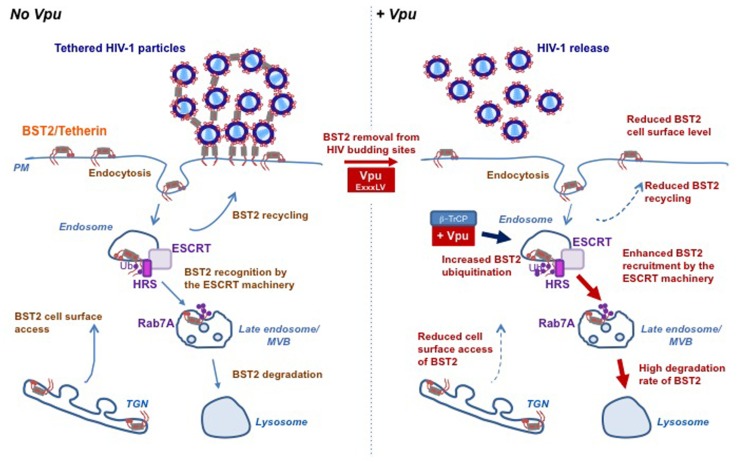
**Vpu subverts the host cell trafficking and ubiquitin machineries to counteract BST2 antiviral activity**. BST2 physically tethers HIV-1 particles on the surface of infected cells preventing their release and further dissemination. The accessory protein Vpu counteracts this restriction by hijacking the host machineries involved in BST2 trafficking and turnover. BST2 cycles between the plasma membrane, the TGN and the endosomes, with a fraction sorted for lysosomal degradation through an ESCRT-mediated pathway (left panel). Vpu counteracts BST2 restriction activity through (i) displacing it from viral assembly sites by a yet-to-be-determined mechanism involving a “dileucine”-like motif located in its cytoplasmic tail and (ii) down-regulating BST2 cell surface expression level and as such restores viral release. Vpu down-regulates cell surface BST2 by slowing down the PM access of internalized and newly synthesized BST2. Vpu-induced BST2 cell surface down regulation is associated with targeting of the restriction factor for lysosomal degradation through an ubiquitin-dependent mechanism that involves the recruitment of SCF^β-TrCP^ E3 ubiquitin ligase complex and the ESCRT machinery. Indeed, through binding to HRS, Vpu enhances the affinity of BST2 for HRS thus accelerating its sorting by the ESCRT machinery for lysosomal degradation. One might propose that through binding with the ESCRT machinery, Vpu may favor the sorting of BST2 at the level of endosomes, thereby reducing its recycling to the plasma membrane (right panel). This figure is adapted from Janvier et al. (2012).

The mechanism whereby Vpu decreases cell surface BST2 expression appears to rely on interference with BST2 intracellular trafficking. BST2 was thought to cycle between the PM, the TGN and the endosomes, with a fraction sorted for lysosomal degradation through an Endosomal Sorting Complexes Required for Transport (ESCRT)-mediated pathway (Rollason et al., 2007; Masuyama et al., 2009; Habermann et al., 2010; Janvier et al., 2011). Internalization of BST2 from the PM occurs through clathrin-coated vesicles, *via* direct binding of the clathrin adaptor complexes AP2 with non-canonical dual tyrosine residues (Y_6_XY_8_) in the cytoplasmic tail of BST2 (Rollason et al., 2007; Masuyama et al., 2009). Binding of BST2 with AP1 complexes regulates its retrieval from the early endosomes back to the TGN (Rollason et al., 2007). Vpu does not increase the rate of BST2 endocytosis but rather slows down the recycling of internalized BST2 back to the PM and inhibits the access of *de novo* synthetized BST2 to the cell surface, thereby decreasing the resupply of BST2 to the PM (Mitchell et al., 2009; Dube et al., 2011; Lau et al., 2011; Schmidt et al., 2011; **Figure 2**).

In some cell types such as T-cells and primary macrophages, antagonism of BST2 by Vpu is not associated with decreased expression of cell surface BST2 (Miyagi et al., 2009; Chu et al., 2012), consistent with the view that Vpu promotes viral release by displacing BST2 from viral budding sites at the PM. Using a sophisticated approach, it has been recently suggested that this function relies on the integrity of a “dileucine”-like motif E_59_VSAL_63_V in the cytoplasmic tail of Vpu (McNatt et al., 2013), first reported to be essential for Vpu to down-regulate CD4 and BST2 expression and counteract BST2 antiviral activity (Hill et al., 2010; Kueck and Neil, 2012). How this motif contributes to these functions is, however unclear. Vpu EXXXLV motif fits the consensus dileucine – based sorting signal (D/E)XXXL(L/I/M) that mediates binding to the AP complexes. However, evidence for an interaction of Vpu with the AP complexes or a direct contribution of these complexes in Vpu’s functions could not be demonstrated (Kueck and Neil, 2012).

### VPU-MEDIATED DEGRADATION OF BST2

In some cell types, Vpu-induced down-regulation of cell surface BST2 is associated with enhanced targeting of the cellular protein to the degradation pathway (Gupta et al., 2009a; Mitchell et al., 2009). This process is ubiquitin-dependent and requires the recruitment of the SCF^β-TrCP^ complex by Vpu *via* its DS_52_GxxS_56_ motif (Douglas et al., 2009; Goffinet et al., 2009; Mangeat et al., 2009; Mitchell et al., 2009; Tokarev et al., 2010). The importance of β-TrCP in Vpu-mediated antagonism of BST2 antiviral activity remains controversial to date (Douglas et al., 2009; Iwabu et al., 2009; Mangeat et al., 2009; Mitchell et al., 2009; Miyagi et al., 2009; Tervo et al., 2011). BST2 undergoes ubiquitination on lysine residues located in its cytoplasmic tail (Pardieu et al., 2010). Interestingly, Vpu increases BST2 ubiquitination on lysine/serine and threonine residues located in its cytoplasmic tail, as is also observed with CD4 (Tokarev et al., 2010). Mutation of these residues reduces Vpu-induced antagonism of BST2. Consistent with this observation, the short isoform of BST2 lacking these residues shows decreased sensitivity to Vpu antagonism (Tokarev et al., 2010; Cocka and Bates, 2012). One study challenged the requirement of the S_3_T_4_S_5_ residues in Vpu-induced ubiquitination of BST2 (Gustin et al., 2012) but no explanation has been proposed for this discrepancy.

Despite the similarity in the molecular mechanisms underlying Vpu-induced ubiquitination and degradation of CD4 and BST2, the fate of both proteins differs. Indeed, it is now well established that Vpu does not target BST2 for proteasomal degradation but induces β-TrCP-dependent lysosomal sorting of BST2 (Douglas et al., 2009; Iwabu et al., 2009; Mitchell et al., 2009). In agreement with this notion, we highlighted a major role of Rab7, a regulator of the endo/lysosomal trafficking, in this process (Caillet et al., 2011), and revealed that Vpu enhances ESCRT-mediated sorting of BST2 for degradation (Janvier et al., 2011). The ESCRT machinery is a set of four hetero-oligomeric protein complexes involved in the sorting of ubiquitinated membrane proteins into vesicles budding into endosomes for their subsequent degradation in the lysosomes. The ESCRT-0 protein HRS (hepatocyte growth factor-regulated tyrosine kinase substrate) coordinates this process by linking ubiquitinated cargoes and the ESCRT-I component TSG101 (Raiborg and Stenmark, 2009). We showed that Vpu-mediated down-regulation of BST2 and viral release require HRS, and unveiled an increased affinity of BST2 for HRS upon Vpu expression (Janvier et al., 2011). One might propose that through binding with HRS and BST2, Vpu accelerates ESCRT-mediated sorting of BST2 to the lysosomes, thereby reducing its recycling to the PM (Janvier et al., 2012; **Figure 2**). Further evidence of the requirement of the ESCRT machinery in Vpu-mediated BST2 degradation was obtained by the characterization of a new component of the ESCRT-I machinery: the protein UBAP1 (Agromayor et al., 2012). Depletion of UBAP1 abolishes Vpu-induced degradation of BST2, but has no impact on Vpu antagonism of BST2 antiviral activity (Agromayor et al., 2012), consistent with the notion that degradation of BST2 is not strictly required for Vpu-mediated antagonism of BST2 (Miyagi et al., 2009; Goffinet et al., 2010).

## VPU AND BST2 VIRAL SENSING ACTIVITY

In addition to its activity as a restriction factor of viral release, BST2 has been recently characterized as an innate immune sensor for HIV (Cocka and Bates, 2012; Galao et al., 2012; Tokarev et al., 2013). In 2003, BST2 was first reported to stimulate the activity of the NF-κB family of transcription factors, using a whole-genome cDNA screen (Matsuda et al., 2003). Recent studies further revealed that tethered HIV particles increase BST2 signaling activity, resulting in enhanced production of pro-inflammatory stimuli, consistent with a role of BST2 as a sensor for assembled viruses (Cocka and Bates, 2012; Galao et al., 2012; Tokarev et al., 2013). This function seems separable from its activity as an inhibitor of viral release and relies on the integrity of the non-canonical dual tyrosine residues (Y_6_XY_8_) regulating its trafficking (Galao et al., 2012; Tokarev et al., 2013). Whether BST2 trafficking is relevant for its signaling activity is unclear. BST2 was proposed to activate the “canonical” NF-κB pathway through engaging mediators of this pathway *via* its tyrosine motif (Galao et al., 2012; Tokarev et al., 2013). Regulation of this pathway depends on the activation of the transforming growth factor-β-activated kinase-1 (TAK1)/TAK1-binding protein 1 and 2 (TAB1 and TAB2) complex, through poly-ubiquitination by E3 ligases of the TNF receptor-associated factors (TRAFs) family (Skaug et al., 2009). Interestingly, BST2 was shown to co-immunoprecipitate with TRAF2, TRAF6 as well as the TAK1/TAB1 complex (Galao et al., 2012; Tokarev et al., 2013).

Vpu was shown to counteract BST2 signaling activity through a β-TrCP-dependent mechanism (Tokarev et al., 2013). Interestingly, over a decade ago, Vpu was shown to sequester β-TrCP away from its substrates and inhibit NF-κB activation by interfering with β-TrCP-mediated degradation of the NF-κB inhibitor IκB (Bour et al., 2001; Besnard-Guerin et al., 2004). Whether this mechanism accounts solely for Vpu-induced inhibition of BST2 signaling activity requires further investigation. Furthermore, many questions remain regarding how viral expression triggers BST2 sensing activity.

## CONCLUDING REMARKS

Over the past decade, Vpu has emerged as an important asset for viral egress and evasion from the host antiviral mechanisms. Although tremendous progress has been made toward understanding the mechanisms underlying Vpu’s functions, many questions remain regarding how this protein contributes to viral pathogenesis. Vpu’s contribution to viral immune evasion relies on its ability to alter the trafficking of its targets by subverting cellular machineries involved this process. However, despite some similarities in the mechanisms involved, major differences have been reported regarding the site of action of Vpu as well as the fate of its targets in cells. Further characterization of the mechanisms controlling Vpu expression and distribution in cells as well as the interplay with its targets and the host cell machineries might contribute to explain these pleiotropic effects. In keeping with this line of thought, another fascinating aspect worthy of further investigation is the role of Vpu with regards to BST2, in cell-to-cell transmission of HIV through virological synapses. So far, conflicting results have been obtained regarding the impact of both proteins on this process, but intriguingly, they have underlined a multifaceted role of BST2 in HIV pathogenesis (Schubert et al., 1995; Gummuluru et al., 2000; Casartelli et al., 2010; Jolly et al., 2010). Adding to this complexity, BST2 was recently described to act as a host sensor of assembled viruses. Therefore, a more detailed characterization of BST2’s functions in cells as well as its interplay with Vpu would contribute to better comprehend the role of both proteins in viral egress and dissemination. Addressing all these questions might provide important insights into AIDS pathogenesis and contribute to the future development of therapeutic strategies.

## AUTHOR CONTRIBUTIONS

Nicolas Roy and Grégory Pacini conceived the figures and contributed to the writing of the manuscript; Clarisse Berlioz-Torrent edited the manuscript; Katy Janvier wrote the manuscript and contributed to the drawing of the figures.

## Conflict of Interest Statement

The authors declare that the research was conducted in the absence of any commercial or financial relationships that could be construed as a potential conflict of interest.
